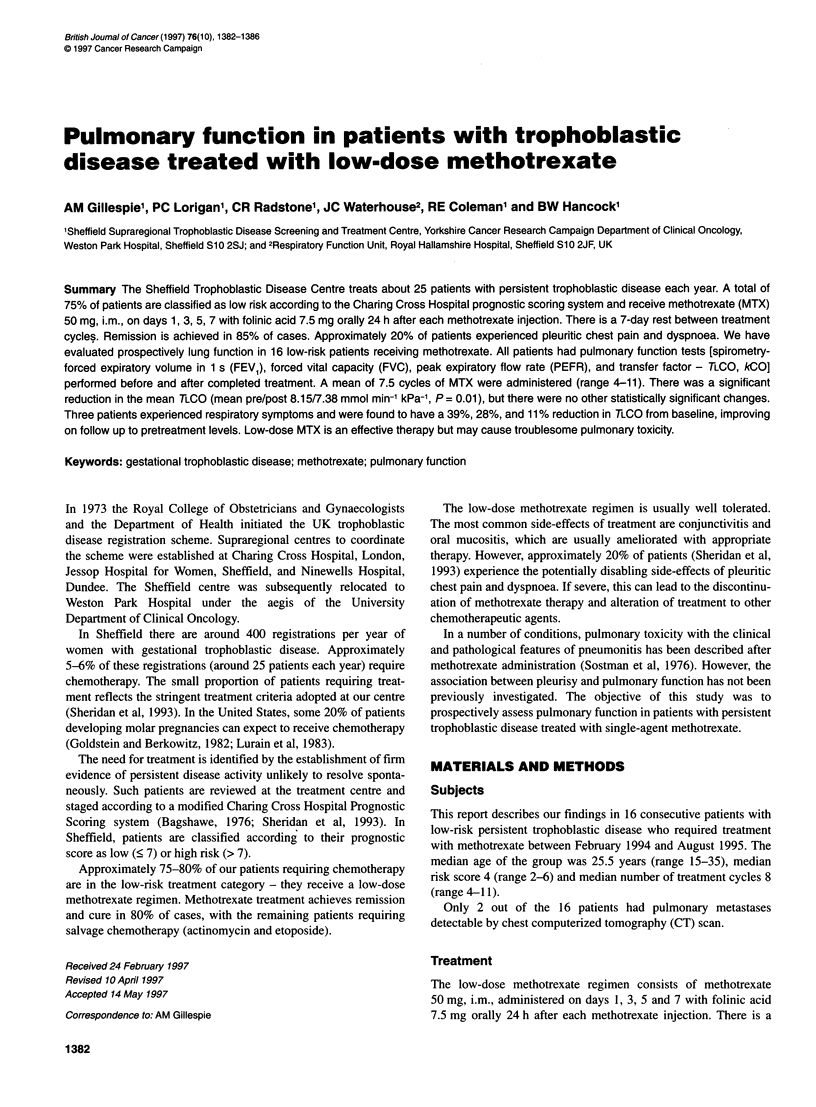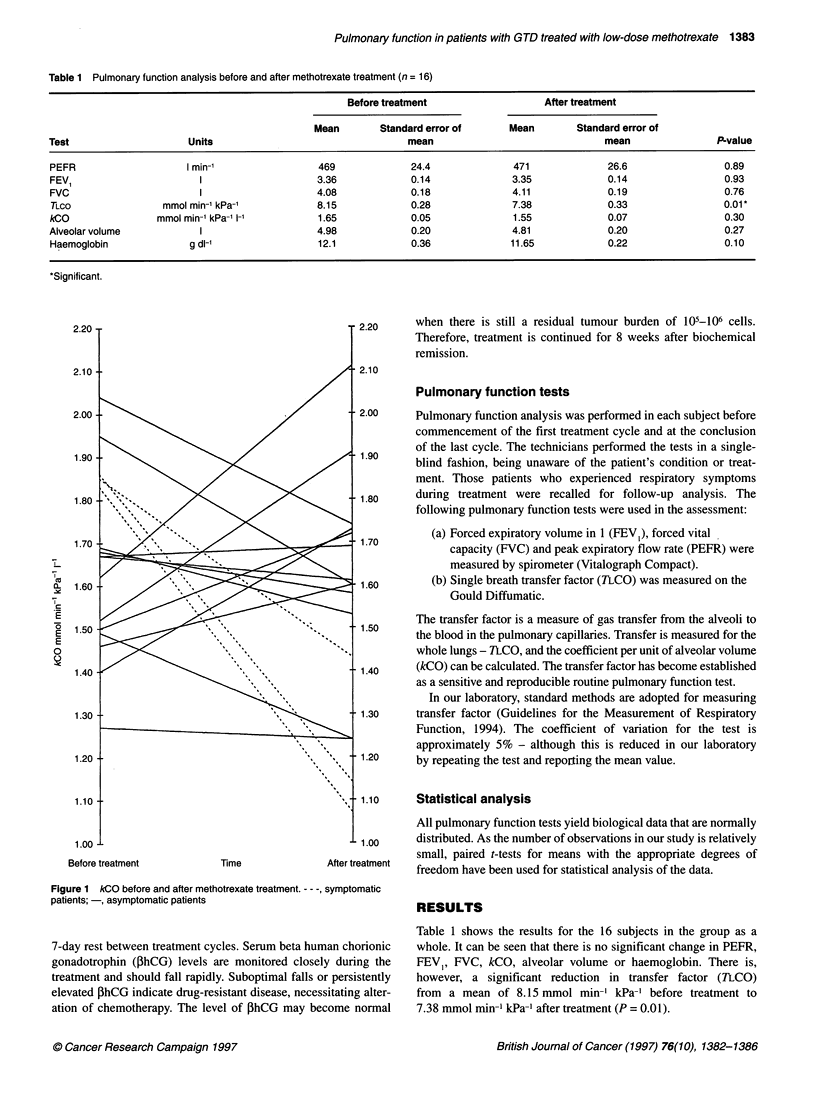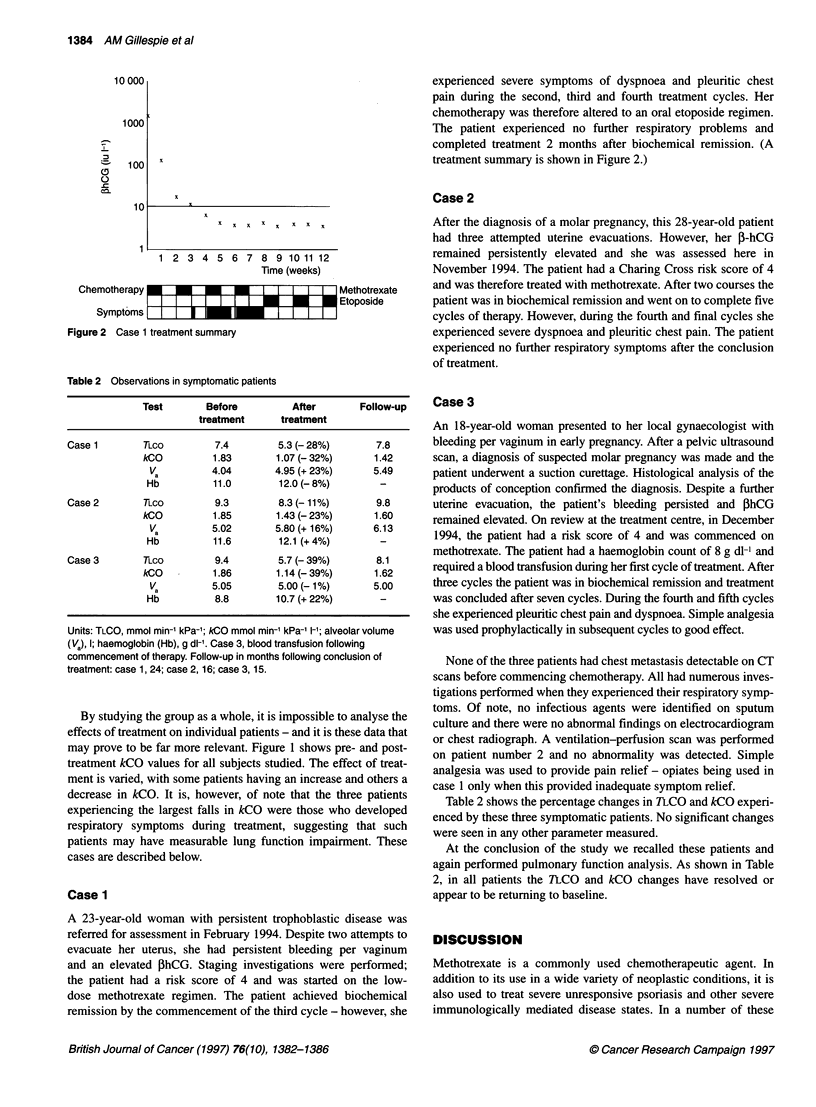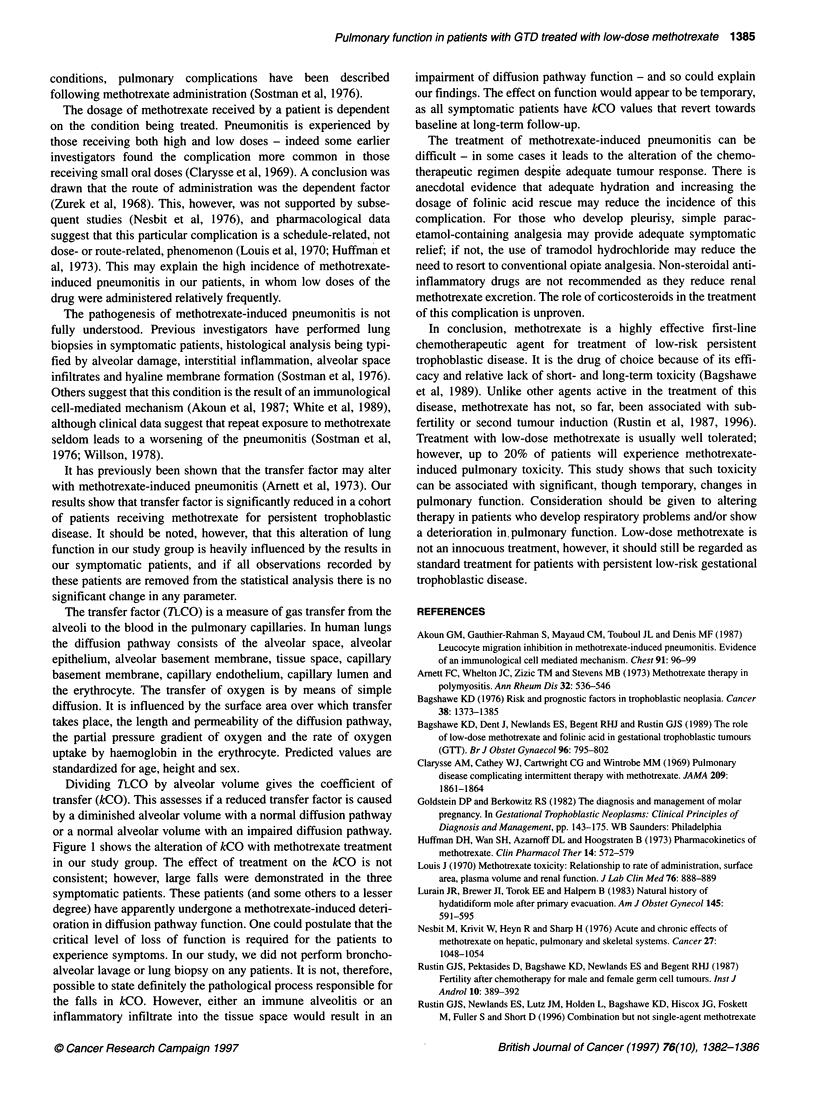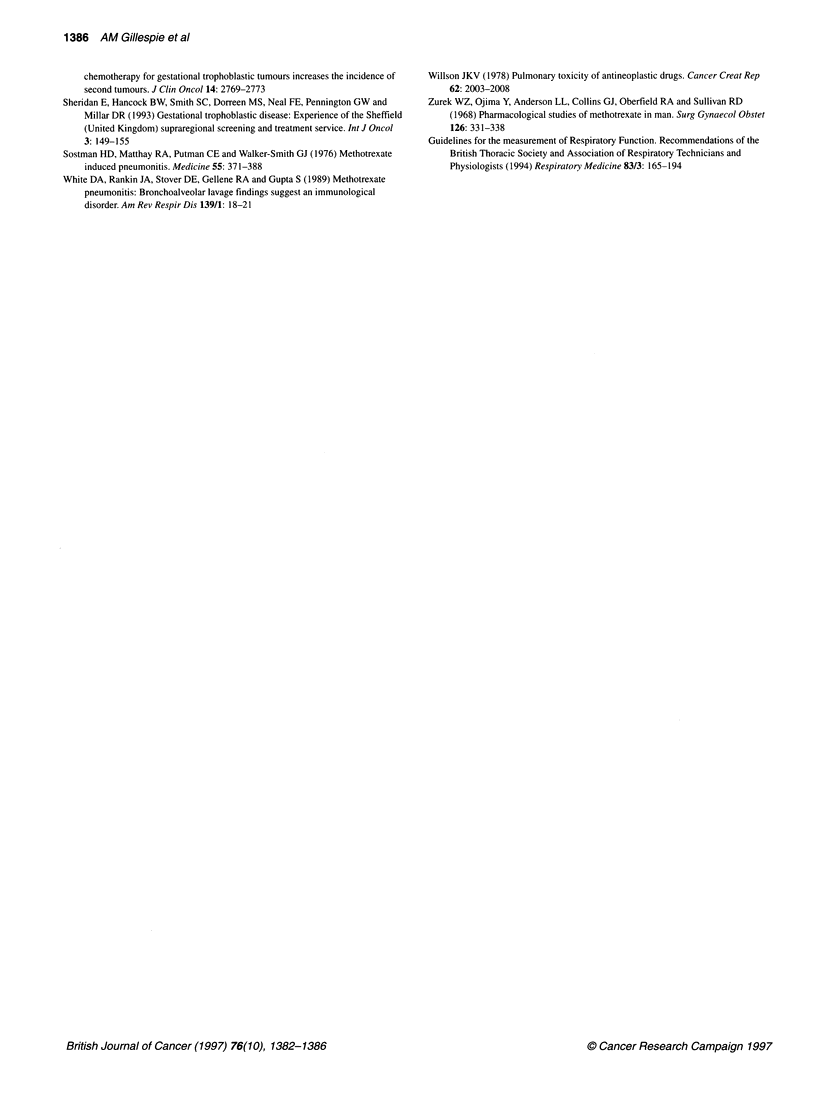# Pulmonary function in patients with trophoblastic disease treated with low-dose methotrexate.

**DOI:** 10.1038/bjc.1997.564

**Published:** 1997

**Authors:** A. M. Gillespie, P. C. Lorigan, C. R. Radstone, J. C. Waterhouse, R. E. Coleman, B. W. Hancock

**Affiliations:** Sheffield Supraregional Trophoblastic Disease Screening and Treatment Centre, Yorkshire Cancer Research Campaign Department of Clinical Oncology, Weston Park Hospital, UK.

## Abstract

The Sheffield Trophoblastic Disease Centre treats about 25 patients with persistent trophoblastic disease each year. A total of 75% of patients are classified as low risk according to the Charing Cross Hospital prognostic scoring system and receive methotrexate (MTX) 50 mg, i.m., on days 1, 3, 5, 7 with folinic acid 7.5 mg orally 24 h after each methotrexate injection. There is a 7-day rest between treatment cycles. Remission is achieved in 85% of cases. Approximately 20% of patients experienced pleuritic chest pain and dyspnoea. We have evaluated prospectively lung function in 16 low-risk patients receiving methotrexate. All patients had pulmonary function tests [spirometry-forced expiratory volume in 1 s (FEV1), forced vital capacity (FVC), peak expiratory flow rate (PEFR), and transfer factor - TLCO, kCO] performed before and after completed treatment. A mean of 7.5 cycles of MTX were administered (range 4-11). There was a significant reduction in the mean TLCO (mean pre/post 8.15/7.38 mmol min-1 kPa-1, P = 0.01), but there were no other statistically significant changes. Three patients experienced respiratory symptoms and were found to have a 39%, 28%, and 11% reduction in TLCO from baseline, improving on follow up to pretreatment levels. Low-dose MTX is an effective therapy but may cause troublesome pulmonary toxicity.


British Journal of Cancer (1997) 76(10), 1382–1386
© 1997 Cancer Research Campaign

### Pulmonary function in patients with trophoblastic disease treated with low-dose methotrexate

**AM Gillespie[1], PC Lorigan[1], CR Radstone[1], JC Waterhouse[2], RE Coleman[1] and BW Hancock[1]**

[1]Sheffield Supraregional Trophoblastic Disease Screening and Treatment Centre, Yorkshire Cancer Research Campaign Department of Clinical Oncology, Weston Park Hospital, Sheffield S10 2SJ; and [2]Respiratory Function Unit, Royal Hallamshire Hospital, Sheffield S10 2JF, UK

**Summary** The Sheffield Trophoblastic Disease Centre treats about 25 patients with persistent trophoblastic disease each year. A total of 75% of patients are classified as low risk according to the Charing Cross Hospital prognostic scoring system and receive methotrexate (MTX) 50 mg, i.m., on days 1, 3, 5, 7 with folinic acid 7.5 mg orally 24 h after each methotrexate injection. There is a 7-day rest between treatment cycles. Remission is achieved in 85% of cases. Approximately 20% of patients experienced pleuritic chest pain and dyspnoea. We have evaluated prospectively lung function in 16 low-risk patients receiving methotrexate. All patients had pulmonary function tests [spirometry-forced expiratory volume in 1 s ($FEV_1$), forced vital capacity (FVC), peak expiratory flow rate (PEFR), and transfer factor – *T*LCO, *k*CO] performed before and after completed treatment. A mean of 7.5 cycles of MTX were administered (range 4–11). There was a significant reduction in the mean *T*LCO (mean pre/post 8.15/7.38 mmol $min^{-1}$ $kPa^{-1}$, $P = 0.01$), but there were no other statistically significant changes. Three patients experienced respiratory symptoms and were found to have a 39%, 28%, and 11% reduction in *T*LCO from baseline, improving on follow up to pretreatment levels. Low-dose MTX is an effective therapy but may cause troublesome pulmonary toxicity.

**Keywords:** gestational trophoblastic disease; methotrexate; pulmonary function

In 1973 the Royal College of Obstetricians and Gynaecologists and the Department of Health initiated the UK trophoblastic disease registration scheme. Supraregional centres to coordinate the scheme were established at Charing Cross Hospital, London, Jessop Hospital for Women, Sheffield, and Ninewells Hospital, Dundee. The Sheffield centre was subsequently relocated to Weston Park Hospital under the aegis of the University Department of Clinical Oncology.

In Sheffield there are around 400 registrations per year of women with gestational trophoblastic disease. Approximately 5–6% of these registrations (around 25 patients each year) require chemotherapy. The small proportion of patients requiring treatment reflects the stringent treatment criteria adopted at our centre (Sheridan et al, 1993). In the United States, some 20% of patients developing molar pregnancies can expect to receive chemotherapy (Goldstein and Berkowitz, 1982; Lurain et al, 1983).

The need for treatment is identified by the establishment of firm evidence of persistent disease activity unlikely to resolve spontaneously. Such patients are reviewed at the treatment centre and staged according to a modified Charing Cross Hospital Prognostic Scoring system (Bagshawe, 1976; Sheridan et al, 1993). In Sheffield, patients are classified according to their prognostic score as low ($\leq 7$) or high risk ($> 7$).

Approximately 75–80% of our patients requiring chemotherapy are in the low-risk treatment category – they receive a low-dose methotrexate regimen. Methotrexate treatment achieves remission and cure in 80% of cases, with the remaining patients requiring salvage chemotherapy (actinomycin and etoposide).

*Received 24 February 1997*
*Revised 10 April 1997*
*Accepted 14 May 1997*

*Correspondence to:* AM Gillespie

The low-dose methotrexate regimen is usually well tolerated. The most common side-effects of treatment are conjunctivitis and oral mucositis, which are usually ameliorated with appropriate therapy. However, approximately 20% of patients (Sheridan et al, 1993) experience the potentially disabling side-effects of pleuritic chest pain and dyspnoea. If severe, this can lead to the discontinuation of methotrexate therapy and alteration of treatment to other chemotherapeutic agents.

In a number of conditions, pulmonary toxicity with the clinical and pathological features of pneumonitis has been described after methotrexate administration (Sostman et al, 1976). However, the association between pleurisy and pulmonary function has not been previously investigated. The objective of this study was to prospectively assess pulmonary function in patients with persistent trophoblastic disease treated with single-agent methotrexate.

### MATERIALS AND METHODS

### Subjects

This report describes our findings in 16 consecutive patients with low-risk persistent trophoblastic disease who required treatment with methotrexate between February 1994 and August 1995. The median age of the group was 25.5 years (range 15–35), median risk score 4 (range 2–6) and median number of treatment cycles 8 (range 4–11).

Only 2 out of the 16 patients had pulmonary metastases detectable by chest computerized tomography (CT) scan.

### Treatment

The low-dose methotrexate regimen consists of methotrexate 50 mg, i.m., administered on days 1, 3, 5 and 7 with folinic acid 7.5 mg orally 24 h after each methotrexate injection. There is a

**Table 1** Pulmonary function analysis before and after methotrexate treatment ($n$ = 16)

| Test | Units | Before treatment Mean | Before treatment Standard error of mean | After treatment Mean | After treatment Standard error of mean | *P*-value |
|---|---|---|---|---|---|---|
| PEFR | l min$^{-1}$ | 469 | 24.4 | 471 | 26.6 | 0.89 |
| $FEV_1$ | l | 3.36 | 0.14 | 3.35 | 0.14 | 0.93 |
| FVC | l | 4.08 | 0.18 | 4.11 | 0.19 | 0.76 |
| *T*LCO | mmol min$^{-1}$ kPa$^{-1}$ | 8.15 | 0.28 | 7.38 | 0.33 | 0.01* |
| *k*CO | mmol min$^{-1}$ kPa$^{-1}$ l$^{-1}$ | 1.65 | 0.05 | 1.55 | 0.07 | 0.30 |
| Alveolar volume | l | 4.98 | 0.20 | 4.81 | 0.20 | 0.27 |
| Haemoglobin | g dl$^{-1}$ | 12.1 | 0.36 | 11.65 | 0.22 | 0.10 |

*Significant.

**Figure 1** *k*CO before and after methotrexate treatment. - - -, symptomatic patients; —, asymptomatic patients

7-day rest between treatment cycles. Serum beta human chorionic gonadotrophin (βhCG) levels are monitored closely during the treatment and should fall rapidly. Suboptimal falls or persistently elevated βhCG indicate drug-resistant disease, necessitating alteration of chemotherapy. The level of βhCG may become normal when there is still a residual tumour burden of $10^5$–$10^6$ cells. Therefore, treatment is continued for 8 weeks after biochemical remission.

### Pulmonary function tests

Pulmonary function analysis was performed in each subject before commencement of the first treatment cycle and at the conclusion of the last cycle. The technicians performed the tests in a single-blind fashion, being unaware of the patient's condition or treatment. Those patients who experienced respiratory symptoms during treatment were recalled for follow-up analysis. The following pulmonary function tests were used in the assessment:

(a) Forced expiratory volume in 1 ($FEV_1$), forced vital capacity (FVC) and peak expiratory flow rate (PEFR) were measured by spirometer (Vitalograph Compact).
(b) Single breath transfer factor (*T*LCO) was measured on the Gould Diffumatic.

The transfer factor is a measure of gas transfer from the alveoli to the blood in the pulmonary capillaries. Transfer is measured for the whole lungs – *T*LCO, and the coefficient per unit of alveolar volume (*k*CO) can be calculated. The transfer factor has become established as a sensitive and reproducible routine pulmonary function test.

In our laboratory, standard methods are adopted for measuring transfer factor (Guidelines for the Measurement of Respiratory Function, 1994). The coefficient of variation for the test is approximately 5% – although this is reduced in our laboratory by repeating the test and reporting the mean value.

### Statistical analysis

All pulmonary function tests yield biological data that are normally distributed. As the number of observations in our study is relatively small, paired *t*-tests for means with the appropriate degrees of freedom have been used for statistical analysis of the data.

### RESULTS

Table 1 shows the results for the 16 subjects in the group as a whole. It can be seen that there is no significant change in PEFR, $FEV_1$, FVC, *k*CO, alveolar volume or haemoglobin. There is, however, a significant reduction in transfer factor (*T*LCO) from a mean of 8.15 mmol min$^{-1}$ kPa$^{-1}$ before treatment to 7.38 mmol min$^{-1}$ kPa$^{-1}$ after treatment ($P$ = 0.01).

**Figure 2** Case 1 treatment summary

**Table 2** Observations in symptomatic patients

| | Test | Before treatment | After treatment | Follow-up |
|---|---|---|---|---|
| Case 1 | *T*LCO | 7.4 | 5.3 (– 28%) | 7.8 |
| | *k*CO | 1.83 | 1.07 (– 32%) | 1.42 |
| | $V_a$ | 4.04 | 4.95 (+ 23%) | 5.49 |
| | Hb | 11.0 | 12.0 (– 8%) | – |
| Case 2 | *T*LCO | 9.3 | 8.3 (– 11%) | 9.8 |
| | *k*CO | 1.85 | 1.43 (– 23%) | 1.60 |
| | $V_a$ | 5.02 | 5.80 (+ 16%) | 6.13 |
| | Hb | 11.6 | 12.1 (+ 4%) | – |
| Case 3 | *T*LCO | 9.4 | 5.7 (– 39%) | 8.1 |
| | *k*CO | 1.86 | 1.14 (– 39%) | 1.62 |
| | $V_a$ | 5.05 | 5.00 (– 1%) | 5.00 |
| | Hb | 8.8 | 10.7 (+ 22%) | – |

Units: TLCO, mmol $min^{-1}$ $kPa^{-1}$; *k*CO mmol $min^{-1}$ $kPa^{-1}$ $l^{-1}$; alveolar volume ($V_a$), l; haemoglobin (Hb), g $dl^{-1}$. Case 3, blood transfusion following commencement of therapy. Follow-up in months following conclusion of treatment: case 1, 24; case 2, 16; case 3, 15.

By studying the group as a whole, it is impossible to analyse the effects of treatment on individual patients – and it is these data that may prove to be far more relevant. Figure 1 shows pre- and post-treatment *k*CO values for all subjects studied. The effect of treatment is varied, with some patients having an increase and others a decrease in *k*CO. It is, however, of note that the three patients experiencing the largest falls in *k*CO were those who developed respiratory symptoms during treatment, suggesting that such patients may have measurable lung function impairment. These cases are described below.

### Case 1

A 23-year-old woman with persistent trophoblastic disease was referred for assessment in February 1994. Despite two attempts to evacuate her uterus, she had persistent bleeding per vaginum and an elevated βhCG. Staging investigations were performed; the patient had a risk score of 4 and was started on the low-dose methotrexate regimen. The patient achieved biochemical remission by the commencement of the third cycle – however, she experienced severe symptoms of dyspnoea and pleuritic chest pain during the second, third and fourth treatment cycles. Her chemotherapy was therefore altered to an oral etoposide regimen. The patient experienced no further respiratory problems and completed treatment 2 months after biochemical remission. (A treatment summary is shown in Figure 2.)

### Case 2

After the diagnosis of a molar pregnancy, this 28-year-old patient had three attempted uterine evacuations. However, her β-hCG remained persistently elevated and she was assessed here in November 1994. The patient had a Charing Cross risk score of 4 and was therefore treated with methotrexate. After two courses the patient was in biochemical remission and went on to complete five cycles of therapy. However, during the fourth and final cycles she experienced severe dyspnoea and pleuritic chest pain. The patient experienced no further respiratory symptoms after the conclusion of treatment.

### Case 3

An 18-year-old woman presented to her local gynaecologist with bleeding per vaginum in early pregnancy. After a pelvic ultrasound scan, a diagnosis of suspected molar pregnancy was made and the patient underwent a suction curettage. Histological analysis of the products of conception confirmed the diagnosis. Despite a further uterine evacuation, the patient's bleeding persisted and βhCG remained elevated. On review at the treatment centre, in December 1994, the patient had a risk score of 4 and was commenced on methotrexate. The patient had a haemoglobin count of 8 g $dl^{-1}$ and required a blood transfusion during her first cycle of treatment. After three cycles the patient was in biochemical remission and treatment was concluded after seven cycles. During the fourth and fifth cycles she experienced pleuritic chest pain and dyspnoea. Simple analgesia was used prophylactically in subsequent cycles to good effect.

None of the three patients had chest metastasis detectable on CT scans before commencing chemotherapy. All had numerous investigations performed when they experienced their respiratory symptoms. Of note, no infectious agents were identified on sputum culture and there were no abnormal findings on electrocardiogram or chest radiograph. A ventilation–perfusion scan was performed on patient number 2 and no abnormality was detected. Simple analgesia was used to provide pain relief – opiates being used in case 1 only when this provided inadequate symptom relief.

Table 2 shows the percentage changes in *T*LCO and *k*CO experienced by these three symptomatic patients. No significant changes were seen in any other parameter measured.

At the conclusion of the study we recalled these patients and again performed pulmonary function analysis. As shown in Table 2, in all patients the *T*LCO and *k*CO changes have resolved or appear to be returning to baseline.

### DISCUSSION

Methotrexate is a commonly used chemotherapeutic agent. In addition to its use in a wide variety of neoplastic conditions, it is also used to treat severe unresponsive psoriasis and other severe immunologically mediated disease states. In a number of these

© Cancer Research Campaign 1997

conditions, pulmonary complications have been described following methotrexate administration (Sostman et al, 1976).

The dosage of methotrexate received by a patient is dependent on the condition being treated. Pneumonitis is experienced by those receiving both high and low doses – indeed some earlier investigators found the complication more common in those receiving small oral doses (Clarysse et al, 1969). A conclusion was drawn that the route of administration was the dependent factor (Zurek et al, 1968). This, however, was not supported by subsequent studies (Nesbit et al, 1976), and pharmacological data suggest that this particular complication is a schedule-related, not dose- or route-related, phenomenon (Louis et al, 1970; Huffman et al, 1973). This may explain the high incidence of methotrexate-induced pneumonitis in our patients, in whom low doses of the drug were administered relatively frequently.

The pathogenesis of methotrexate-induced pneumonitis is not fully understood. Previous investigators have performed lung biopsies in symptomatic patients, histological analysis being typified by alveolar damage, interstitial inflammation, alveolar space infiltrates and hyaline membrane formation (Sostman et al, 1976). Others suggest that this condition is the result of an immunological cell-mediated mechanism (Akoun et al, 1987; White et al, 1989), although clinical data suggest that repeat exposure to methotrexate seldom leads to a worsening of the pneumonitis (Sostman et al, 1976; Willson, 1978).

It has previously been shown that the transfer factor may alter with methotrexate-induced pneumonitis (Arnett et al, 1973). Our results show that transfer factor is significantly reduced in a cohort of patients receiving methotrexate for persistent trophoblastic disease. It should be noted, however, that this alteration of lung function in our study group is heavily influenced by the results in our symptomatic patients, and if all observations recorded by these patients are removed from the statistical analysis there is no significant change in any parameter.

The transfer factor (*T*LCO) is a measure of gas transfer from the alveoli to the blood in the pulmonary capillaries. In human lungs the diffusion pathway consists of the alveolar space, alveolar epithelium, alveolar basement membrane, tissue space, capillary basement membrane, capillary endothelium, capillary lumen and the erythrocyte. The transfer of oxygen is by means of simple diffusion. It is influenced by the surface area over which transfer takes place, the length and permeability of the diffusion pathway, the partial pressure gradient of oxygen and the rate of oxygen uptake by haemoglobin in the erythrocyte. Predicted values are standardized for age, height and sex.

Dividing *T*LCO by alveolar volume gives the coefficient of transfer (*k*CO). This assesses if a reduced transfer factor is caused by a diminished alveolar volume with a normal diffusion pathway or a normal alveolar volume with an impaired diffusion pathway. Figure 1 shows the alteration of *k*CO with methotrexate treatment in our study group. The effect of treatment on the *k*CO is not consistent; however, large falls were demonstrated in the three symptomatic patients. These patients (and some others to a lesser degree) have apparently undergone a methotrexate-induced deterioration in diffusion pathway function. One could postulate that the critical level of loss of function is required for the patients to experience symptoms. In our study, we did not perform broncho-alveolar lavage or lung biopsy on any patients. It is not, therefore, possible to state definitely the pathological process responsible for the falls in *k*CO. However, either an immune alveolitis or an inflammatory infiltrate into the tissue space would result in an impairment of diffusion pathway function – and so could explain our findings. The effect on function would appear to be temporary, as all symptomatic patients have *k*CO values that revert towards baseline at long-term follow-up.

The treatment of methotrexate-induced pneumonitis can be difficult – in some cases it leads to the alteration of the chemotherapeutic regimen despite adequate tumour response. There is anecdotal evidence that adequate hydration and increasing the dosage of folinic acid rescue may reduce the incidence of this complication. For those who develop pleurisy, simple paracetamol-containing analgesia may provide adequate symptomatic relief; if not, the use of tramodol hydrochloride may reduce the need to resort to conventional opiate analgesia. Non-steroidal anti-inflammatory drugs are not recommended as they reduce renal methotrexate excretion. The role of corticosteroids in the treatment of this complication is unproven.

In conclusion, methotrexate is a highly effective first-line chemotherapeutic agent for treatment of low-risk persistent trophoblastic disease. It is the drug of choice because of its efficacy and relative lack of short- and long-term toxicity (Bagshawe et al, 1989). Unlike other agents active in the treatment of this disease, methotrexate has not, so far, been associated with subfertility or second tumour induction (Rustin et al, 1987, 1996). Treatment with low-dose methotrexate is usually well tolerated; however, up to 20% of patients will experience methotrexate-induced pulmonary toxicity. This study shows that such toxicity can be associated with significant, though temporary, changes in pulmonary function. Consideration should be given to altering therapy in patients who develop respiratory problems and/or show a deterioration in pulmonary function. Low-dose methotrexate is not an innocuous treatment, however, it should still be regarded as standard treatment for patients with persistent low-risk gestational trophoblastic disease.

### REFERENCES

Akoun GM, Gauthier-Rahman S, Mayaud CM, Touboul JL and Denis MF (1987) Leucocyte migration inhibition in methotrexate-induced pneumonitis. Evidence of an immunological cell mediated mechanism. *Chest* **91**: 96–99

Arnett FC, Whelton JC, Zizic TM and Stevens MB (1973) Methotrexate therapy in polymyositis. *Ann Rheum Dis* **32**: 536–546

Bagshawe KD (1976) Risk and prognostic factors in trophoblastic neoplasia. *Cancer* **38**: 1373–1385

Bagshawe KD, Dent J, Newlands ES, Begent RHJ and Rustin GJS (1989) The role of low-dose methotrexate and folinic acid in gestational trophoblastic tumours (GTT). *Br J Obstet Gynaecol* **96**: 795–802

Clarysse AM, Cathey WJ, Cartwright CG and Wintrobe MM (1969) Pulmonary disease complicating intermittent therapy with methotrexate. *JAMA* **209**: 1861–1864

Goldstein DP and Berkowitz RS (1982) The diagnosis and management of molar pregnancy. In *Gestational Trophoblastic Neoplasms: Clinical Principles of Diagnosis and Management*, pp. 143–175. WB Saunders: Philadelphia

Huffman DH, Wan SH, Azarnoff DL and Hoogstraten B (1973) Pharmacokinetics of methotrexate. *Clin Pharmacol Ther* **14**: 572–579

Louis J (1970) Methotrexate toxicity: Relationship to rate of administration, surface area, plasma volume and renal function. *J Lab Clin Med* **76**: 888–889

Lurain JR, Brewer JI, Torok EE and Halpern B (1983) Natural history of hydatidiform mole after primary evacuation. *Am J Obstet Gynecol* **145**: 591–595

Nesbit M, Krivit W, Heyn R and Sharp H (1976) Acute and chronic effects of methotrexate on hepatic, pulmonary and skeletal systems. *Cancer* **27**: 1048–1054

Rustin GJS, Pektasides D, Bagshawe KD, Newlands ES and Begent RHJ (1987) Fertility after chemotherapy for male and female germ cell tumours. *Inst J Androl* **10**: 389–392

Rustin GJS, Newlands ES, Lutz JM, Holden L, Bagshawe KD, Hiscox JG, Foskett M, Fuller S and Short D (1996) Combination but not single-agent methotrexate

© Cancer Research Campaign 1997

chemotherapy for gestational trophoblastic tumours increases the incidence of second tumours. *J Clin Oncol* **14**: 2769–2773

Sheridan E, Hancock BW, Smith SC, Dorreen MS, Neal FE, Pennington GW and Millar DR (1993) Gestational trophoblastic disease: Experience of the Sheffield (United Kingdom) supraregional screening and treatment service. *Int J Oncol* **3**: 149–155

Sostman HD, Matthay RA, Putman CE and Walker-Smith GJ (1976) Methotrexate induced pneumonitis. *Medicine* **55**: 371–388

White DA, Rankin JA, Stover DE, Gellene RA and Gupta S (1989) Methotrexate pneumonitis: Bronchoalveolar lavage findings suggest an immunological disorder. *Am Rev Respir Dis* **139/1**: 18–21

Willson JKV (1978) Pulmonary toxicity of antineoplastic drugs. *Cancer Creat Rep* **62**: 2003–2008

Zurek WZ, Ojima Y, Anderson LL, Collins GJ, Oberfield RA and Sullivan RD (1968) Pharmacological studies of methotrexate in man. *Surg Gynaecol Obstet* **126**: 331–338

Guidelines for the measurement of Respiratory Function. Recommendations of the British Thoracic Society and Association of Respiratory Technicians and Physiologists (1994) *Respiratory Medicine* **83/3**: 165–194

© Cancer Research Campaign 1997